# Prevalence of metabolic syndrome in patients with schizophrenia, and metabolic changes after 3 months of treatment with antipsychotics - results from a German observational study

**DOI:** 10.1186/1471-244X-11-173

**Published:** 2011-11-01

**Authors:** Susanne Kraemer, Anette Minarzyk, Thomas Forst, Daniel Kopf, Hans-Peter Hundemer

**Affiliations:** 1 Lilly Deutschland GmbH, Medical Department, 61352 Bad Homburg, Werner -Reimers-Str. 2-4, Germany; 2 Institute for Clinical Research IKFE, 55116 Mainz, Parcusstr. 8, Germany; 3 Kath. Marienkrankenhaus GmbH, Geriatrics Clinic, 22087 Hamburg, Alfredstr.9, Germany

## Abstract

**Background:**

This observational study explored the prevalence of metabolic syndrome (MetS) in adult in- and outpatients with untreated or treated schizophrenia at baseline, and month-3 after initiation or switch of antipsychotic treatment.

**Methods:**

MetS-prevalence (AHA/NHLB-definition) was assessed and Clopper-Pearson 95% confidence intervals (CIs) were calculated. Factors associated with MetS were explored through univariate and multivariate logistic regressions (both visits).

**Results:**

MetS-prevalence was 44.3% (CI 39.8;48.9) at baseline and 49.6% (CI 45.0;54.2) at month-3. Previously unmedicated patients showed the lowest baseline MetS-prevalence (24.7%, CI 18.3;32.1). MetS-prevalence was not significantly different, regardless if patients previously received typical or atypical antipsychotics. Increased MetS-risk was associated with somatic comorbidity and non-smoking at both visits, and with non-psychiatric co-medication, male sex, and increased C-reactive protein at month-3.

**Conclusions:**

At baseline, MetS was most prevalent in patients with previous antipsychotic medication. Limited metabolic changes were observed 3 months after switch/initiation of antipsychotic therapy.

**Trial Registration Number:**

ClinicalTrials.gov Identifier: n.a.

## Background

Several studies have reported increased mortality in patients with schizophrenia. Besides higher risks for cancer, respiratory and cerebrovascular disorders, and of death from suicide or homicide, the main cause is cardiovascular disease [1-7]. Even before antipsychotic medication became available in the 1950s, abnormal responses to insulin and diabetes-like glucose tolerance curves [8,9] were observed in psychiatric patients. Planansky and Heilizer [10] reported weight gain already in 1959 in patients treated with chlorpromazine. Thakore et al. [11] found higher BMI (body mass index), WHR (waist/hip ratio), and a threefold amount of intra-abdominal fat (IAF) in untreated schizophrenia patients compared to healthy controls. Further factors associated with schizophrenia, like unhealthy diet patterns [12], smoking [13], lower levels of physical activity and cardiorespiratory fitness [14], and poor living conditions certainly contribute to the finding that these patients, including those on antipsychotics, may have a higher risk to develop metabolic syndrome (MetS) than the general population [1,15,16]. It has been suggested that changes in metabolic parameters in patients treated with antipsychotics may, in part, be genetically determined [17].

MetS is characterized by the coincidence of hypertension, abdominal obesity, impaired lipid metabolism (blood triglycerides, cholesterol) and/or impaired blood glucose regulation. Though the concept of MetS is universally accepted, there is still controversy on the exact pathophysiology, resulting in differing definitions (e.g. by the American Heart Association [18], the National Cholesterol Education Program [19], and the International Diabetes Federation/Word Health Organization [20]).

Nevertheless has the awareness of schizophrenia patients' risk to develop MetS resulted in treatment guidelines which demand the regular monitoring of relevant physical and laboratory parameters; in several countries these are meanwhile regarded clinical standard of care [21,22].

Few data are available so far on the prevalence of MetS in schizophrenia patients in Germany. In our observational study we addressed this gap, assessing the prevalence of MetS at baseline and month-3 of treatment with different antipsychotic medications as well as possible predictors for the development of MetS.

## Methods

### Study design

This was a prospective, 3-month, multi-center, disease-oriented, observational study conducted in Germany from September 2006 to April 2008. Eligible were in- and outpatients (≥ 18 years) diagnosed with schizophrenia according to ICD-10 criteria, who either entered the study untreated and were initiated on antipsychotic therapy, or were on antipsychotic treatment and needed to be switched to a new primary medication (initiation/change of medication at baseline). Additionally, routine blood samples had to be scheduled for these patients at baseline and month-3 irrespective of the study. Due to the observational design, no further clinical in- or exclusion criteria were specified, treatment decisions were entirely left to the discretion of investigators and patients.

The study was approved by the responsible ethical review board. Written informed consent for the release of medical data was obtained from all patients according to local regulations. As the German Society of Psychiatry, Psychotherapy and Neurology [21] recommends metabolic screening for all patients with schizophrenia, referring to the *Consensus Statement of the American Diabetes Association *[23], blood tests are considered standard of care in schizophrenia treatment in Germany. Therefore the ethical review board consented that drawing blood samples did not interfere with the observational design of the study.

Our primary research objective was to assess the prevalence of MetS, as defined by the National Cholesterol Education Program, Adult Treatment Panel III in 2001 (NCEP-ATP III) [19] and the American Heart Association/National Heart, Lung and Blood Institute in 2005 (AHA/NHLB) [18], in a German cohort of patients with schizophrenia. The details of both definitions are given in Table 1. As a secondary outcome, we compared MetS-prevalence at baseline and after three months of treatment with the newly prescribed antipsychotic. A further objective was the detection of predictors for the development of the MetS.

**Table 1:** Definitions and reference ranges for metabolic syndrome according to NCEP-ATP III and AHA/NHLB

Risk factor	Defining measure NCEP-ATP III	Defining measure AHA/NHLB
Abdominal obesity (waist circumference)		

Men	> 102 cm	≥ 102 cm

Women	> 88 cm	≥ 88 cm

Triglycerides	≥ 150 mg/dL	≥ 150 mg/dL or on drug treatment for elevated triglycerides

High density lipoprotein (HDL)		

Men	< 40 mg/dL	< 40 mg/dL or on drug treatment for reduced HDL-cholesterol

Women	< 50 mg/dL	< 50 mg/dL or on drug treatment for reduced HDL-cholesterol

Blood pressure	Systolic ≥ 130 or diastolic ≥ 85 mmHg	Systolic ≥ 130 or diastolic ≥ 85 mmHg or on antihypertensive medication

Fasting glucose	≥ 110 mg/dL	≥ 100 mg/dL or on antidiabetic medication

Abbreviations: AHA/NHLB = American Heart Association/National Heart, Lung and Blood Institute; NCEP-ATP III = National Cholesterol Education Program, Adult Treatment Panel 3rd report

According to both definitions, a diagnosis of metabolic syndrome is established if at least three of the above risk factors are present.

Patients were documented at baseline and at month-3. At baseline, patient demographics and characteristics were recorded. At both visits, vital and physical parameters were collected, and fasting blood samples were drawn and analyzed. Apart from the blood levels of high-density lipoprotein (HDL) cholesterol, triglycerides, and glucose, which were needed to diagnose MetS, we determined C-reactive protein (CRP) [24,25]) as an additional indicator of cardiovascular risk, and HbA_1c _(glycated hemoglobin), to assess long term glucose regulation [26].

Blood samples were analyzed in a central laboratory, which applied test reference ranges (i.e. normal ranges) as per Table 2.

**Table 2:** Test reference ranges applied for blood samples

Parameter	Range
HbA_1c _(%)	4 to 6
Triglycerides (mg/dL)	9 to 150
HDL - Cholesterol (mg/dL)	40 to 150
Glucose (mg/dL)	70 to 115
CRP (mg/L)	0 to 3

Abbreviations: HDL = High density lipoprotein; CRP = C-reactive protein, HbA_1c _= glycated hemoglobin.

For assessment of disease severity, the Clinical Global Impression - Severity scale (CGI-S), which rates the severity of the patient's illness on a 7-point scale (1 = normal to 7 = extremely ill) was used at both visits [27].

### Sample size considerations and statistical analysis

The sample size was designed to reach 2.5% precision for the estimate of MetS prevalence rate - i.e. the 95% confidence interval bounds within estimated rate ± 2.5% ($1.96x\sqrt{\frac{\hat{p}\left(1-\hat{p}\right)}{n}=}0.025$) - and assuming a prevalence rate around 41%, based on results of the CATIE study [28]. This yielded a first estimate of 1486 patients, further adjusted accounting for 25% of drop outs. We finally aimed to enroll 1900 patients.

Statistical analyses were performed on two sets: (a) the full analysis set (FAS), including all patients meeting the entry criteria, and (b) the complete metabolic data set (CMD), comprising all patients with a full set of metabolic data for both visits, who did not change their antipsychotic treatment during the course of the study.

Primary analyses were conducted on the FAS, with subgroups formed according to the antipsychotic treatment they received within 6 months prior to baseline (Prev-AP = previous antipsychotic treatment cohorts).

The evaluations of the secondary outcomes were performed on the CMD-set, with subgroups formed according to the treatment patients received after baseline (New-AP = new antipsychotic treatment cohorts). In both sets, compounds which were less frequently prescribed had to be grouped to reach large enough cohorts for reasonable statistical evaluation.

Patient demographics and characteristics, physical, vital and laboratory parameters were described by standard summary statistics and used to determine the presence of MetS at baseline and at month-3.

Clopper-Pearson exact 95% confidence intervals (CI) relating to MetS prevalence were calculated for both sets of antipsychotic treatment cohorts (Prev-AP, FAS, and New-AP, CMD-Set).

The association between the presence of MetS and possible risk factors for its development was analyzed for each visit separately, through univariate and multi-variable forward selection logistic models (CMD-set). Candidate covariates entered in the forward selection process were not pre-screened based on the results of univariate analyses, all of them were considered. The significance level (chi-square score test) for the forward selection process was set to ≤0.1. No interaction was considered. Odds Ratios (OR) were estimated together with their asymptotic Wald 95% confidence interval. For continuous factors ORs relate to an increase by 1 unit. Tested covariates (both visits) included: age, sex, time since first symptoms, any concomitant somatic diseases (yes/no), any concomitant non-psychiatric medication at baseline (yes/no), Prev-AP cohort (reference category: Prev-None), active smoker (yes/no), CGI-S score at baseline, CRP ≥ 3 mg/L (yes/no), and HbA_1c _≥ 6.5% (yes/no).

## Results

### Patient disposition and baseline characteristics

**Figure 1:**
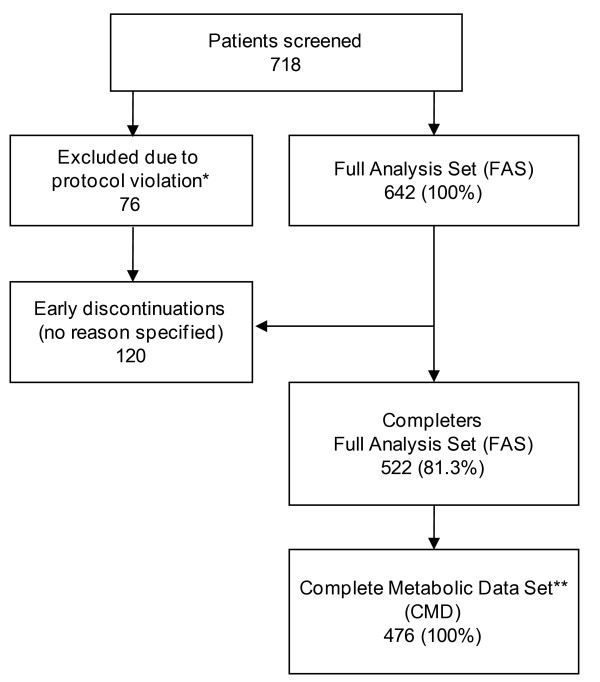
**Patient disposition**. * Time span between baseline visit and blood draw > 3 weeks. ** Patients with complete metabolic data sets for both visits, who did not change antipsychotic treatment during the course of the study.

Only 718 patients could be documented at 162 investigational sites within the recruitment period. Figure 1 displays the details of patient disposition.

Table 3 shows the distribution of patients in the treatment cohorts.

**Table 3:** Patient distribution in treatment cohorts, Prev-AP FAS and New-AP CMD-set

Cohorts Prev-AP, FAS (N = 642)		N (%)
Prev-Olz	previous olanzapine monotherapy	62 (9.7%)
Prev-Risp	previous risperidone monotherapy	67 (10.4%)
Prev-Quet	previous quetiapine monotherapy	49 (7.6%)
Prev-Atyp	previous other atypical antipsychotic monotherapy (amisulpride, aripiprazoleclozapine, ziprasidone, paliperidone)	103 (16.0%)
Prev-Typ	previous typical antipsychotics	90 (14.0%)
Prev-Comb	any previous combination therapy	109 (17.0%)
Prev-None	not treated with antipsychotics within6 months prior to study entry	162 (25.2%)

Cohorts New-AP, CMD-set (N = 476)		N (%)

New-Olz	new olanzapine monotherapy	206 (43.3%)
New-Risp	new risperidone monotherapy	69 (14.5%)
New-Quet	new quetiapine monotherapy	33 (6.9%)
New-Atyp	new other atypical antipsychotic monotherapy (amisulpride, aripiprazoleclozapine, ziprasidone, paliperidone)	72 (15.1%)
New-Typ	new typical antipsychotic	16 (3.4%)
New-Comb	new combination therapy (any combination)	80 (16.8%)

Abbreviations: CMD = complete metabolic data; FAS = full analysis set; New-AP = new antipsychotic treatment cohort; Prev-AP = previous antipsychotic treatment cohort;

The age ranged between 18 and 86 years, with upper and lower quartiles of 36 and 54 years. Women had a mean age of 47.3 ± 13.1 years, for men it was 43.1 ± 13.1 years. A mean waist circumference of 103.5 ± 16.0 cm for men, and 95.6 ± 17.5 cm for women indicated overweight in a considerable proportion of patients. Prev-None was the only cohort with a mean BMI near to normal range (25.3 kg/m²). The mean time since first diagnosis was 9 years, ranging from 0 to 51 years. Baseline characteristics in the overall CMD-set resembled those observed in the FAS. For details on demographics and baseline characteristics of both sets of treatment cohorts, see Table 4 and Table 5.

**Table 4:** Patient Demographics and Baseline Characteristics (Prev-AP cohorts)

Prev-AP*, FAS		Age (years)	BMI (kg/m²)	Waist (cm)	SBP (mm/Hg)	DBP (mm/Hg)	CGI-S score		Male	Smokers
Prev-Olz	Mean	42.9	28.9	103.4	131.1	83.6	3.5	N	36	26
(N = 62)	SD	13.9	5.2	17.1	18.0	8.2	1.2	%	58.1	41.9
Prev-Risp	Mean	46.0	28.9	103.4	128.2	83.1	4.1	N	38	30
(N = 67)	SD	13.2	6.2	17.3	12.7	7.4	1.2	%	56.7	44.8
Prev-Quet	Mean	46.2	27.0	100.0	125.9	81.7	3.9	N	24	17
(N = 49)	SD	12.1	4.9	18.2	13.5	8.5	1.2	%	49.0	34.7
Prev-Atyp	Mean	46.7	28.4	101.1	128.1	81.9	4.0	N	50	43
(N = 103)	SD	13.2	5.8	17.2	16.7	9.9	1.2	%	48.5	41.8
Prev-Typ	Mean	49.1	28.4	102.1	129.3	84.0	4.0	N	42	43
(N = 90)	SD	11.9	5.9	18.7	15.9	9.7	1.2	%	46.7	47.8
Prev-Com	Mean	44.5	29.3	103.3	127.0	82.3	3.6	N	58	43
(N = 109)	SD	11.6	5.4	14.7	11.3	8.9	1.2	%	53.2	39.5
Prev-None	Mean	43.0	25.3	91.3	125.0	80.2	4.2	N	69	61
(N = 162)	SD	14.7	4.5	15.1	15.7	9.3	1.0	%	42.6	37.7
Total FAS	Mean	45.2	27.8	99.5	127.4	82.1	3.9	N	317	263
(N = 642)	SD	13.3	5.6	17.2	15.1	9.1	1.2	%	49.4	41.0

Abbreviations: BMI = body mass index; CGI-S = clinical global impression - severity scale; DBP = diastolic blood pressure; FAS = full analysis set; Prev-AP = previous antipsychotic treatment cohort; SBP = systolic blood pressure; SD = standard deviation, Waist = waist circumference

Missing values: BMI 1 (Prev-Comb), waist circumference 1 (Prev-Comb), SBP and DBP 1 (Prev-Risp)

* The time period through which the previous antipsychotic medication had been taken ranged from less than a month up to more than a decade.

**Table 5:** Patient Demographics and Baseline Characteristics (New-AP cohorts)

New-AP, CMD-set		Age (years)	BMI (kg/m²)	Waist (cm)	SBP (mm/Hg)	DBP (mm/Hg)	CGI-S score		Male	Smokers
New-Olz	Mean	46.3	26.6	96.8	126.3	81.6	4.1	N	106	86
(N = 206)	SD	13.5	4.7	17.2	15.2	8.8	1.2	%	51.5	41.8
New-Risp	Mean	45.6	27.5	98.1	128.4	81.0	4.1	N	30	23
(N = 69)	SD	11.6	5.6	15.9	14.0	8.8	0.9	%	43.5	33.3
New-Quet	Mean	48.5	28.6	100.7	125.6	82.5	3.5	N	11	13
(N = 33)	SD	14.2	4.7	13.5	11.4	7.1	1.3	%	33.3	39.4
New-Atyp	Mean	43.7	29.0	103.9	129.1	82.6	3.7	N	38	35
(N = 72)	SD	11.0	6.2	17.7	14.2	9.1	1.1	%	52.8	48.6
New-Typ	Mean	45.6	32.3	111.3	134.6	84.6	4.1	N	11	3
(N = 16)	SD	11.5	7.0	18.8	16.4	7.3	1.5	%	68.8	18.8
New-Com	Mean	46.0	29.5	105.0	127.3	83.2	3.7	N	40	32
(N = 08)	SD	12.8	5.7	15.9	14.5	9.3	1.3	%	50.0	40.0
Total CMD	Mean	45.9	27.9	100.2	127.4	82.1	3.9	N	236	192
(N = 476)	SD	12.7	5.5	17.1	14.6	8.8	1.2	%	49.6	40.3

Abbreviations: BMI = body mass index; CGI-S = clinical global impression - severity scale; CMD = complete metabolic data; DBP = diastolic blood pressure; New-AP = new antipsychotic treatment cohort; SBP = systolic blood pressure; SD = standard deviation, Waist = waist circumference

In the Prev-None cohort 28.4% of the patients reported any concomitant disease (Table 6), whereas the previously treated patients had rates between 29.9% (Pre-Risp) and 41.7% (Pre-Comb). Non-psychiatric comedication was taken by approximately 20% of the patients, mostly antihypertensives (Table 7).

**Table 6:** Pre-existing concomitant somatic diseases* at baseline (in >5% of the patients, Prev-AP, FAS, N = 642)

		Prev-Olz	Prev-Risp	Prev-Quet	Prev-Atyp	Prev-Typ	Prev-Comb	Prev-None	FAS, total
		N = 62	N = 67	N = 49	N = 103	N = 90	N = 109	N = 162	N = 642
Any	n	23	20	15	36	36	45	46	221
	%	37.1	29.9	30.6	35.0	40.0	41.7	28.4	34.5
	
Hypertension	n	17	12	5	18	18	19	18	107
	%	27.4	17.9	10.2	17.5	20.0	17.6	11.1	16.7
	
Lipid disorders	n	4	7	4	4	7	12	5	43
	%	6.5	10.5	8.2	3.9	7.8	11.1	3.1	6.7
	
Diabetes	n	3	2	3	3	8	15	2	36
	%	4.8	3.0	6.1	2.9	8.9	13.9	1.2	5.6
	
Musculoskeletal disorders	n	1	4	2	4	6	9	8	34
	%	1.6	6.0	4.1	3.9	6.7	8.3	4.9	5.3

Abbreviations: FAS = full analysis set; Prev-AP = previous antipsychotic treatment

*pre-specified in data capturing form: diabetes, lipid metabolism disorder, other endocrine or metabolic disorders, liver disease, hypertension, heart and lung disease, gastrointestinal disease, urinary retention, hematological disease, thrombophilia or other coagulopathy, musculoskeletal disorders, neurological disorders, convulsions, kidney disorders, rheumatic disorder, malignant neoplasm/cancer

**Table 7:** Concomitant non-psychiatric medication at baseline (FAS, N = 642)

Medication	n (%)
None	502 (78.44%)
Statins	12 (1.88%)
Other hypolipidemic drugs	8 (1.25%)
Beta-blockers	62 (9.69%)
Diuretics	24 (3.75%)
Ca-antagonists	10 (1.56%)
ACE-inhibitors	32 (5.00%)
Angiotensin-II-antagonists	2 (0.31%)
Other antihypertensive drugs	22 (3.44%)
Insulins	9 (1.41%)
Oral anti-diabetic drugs	23 (3.59%)
Oral corticosteroids	1 (0.16%)
Corticosteroid inhalants	3 (0.47%)

Abbreviations: ACE-inhibitors = angiotensin-converting enzyme inhibitors; FAS = full analysis set

Table 8 shows the proportions of patients (FAS) with blood test values out of the reference range at baseline. Within the Prev-AP cohorts, the percentages for Prev-None were at the lower end for all parameters.

**Table 8:** Laboratory test: patients with values out of the laboratory test reference range at baseline (Prev-AP, FAS, N = 642)

Blood-Test	Limit*		Prev-Olz	Prev-Risp	Prev-Quet	Prev-Atyp	Prev-Typ	Prev-Comb	Prev-None	FAS, total
			N = 62	N = 67	N = 49	N = 103	N = 90	N = 109	N = 162	N = 642
HbA_1c_	≥6%	n	5	4	5	6	18	15	9	62
		%	8.1	6.0	10.2	5.8	20.0	13.8	5.6	9.7

Glucose	≥115 mg/dL	n	5	10	9	16	17	25	8	90
		%	8.1	14.9	18.4	15.7	18.9	23.2	4.9	14.1

Triglyceride	≥150 mg/dL	n	42	32	28	62	47	66	60	337
		%	67.7	47.8	57.1	60.2	52.2	60.6	37.0	52.5

HDL-Cholesterol	≤40 mg/dL	n	9	9	10	12	10	12	11	73
		%	14.5	13.4	20.4	11.7	11.1	11.0	6.8	11.4

C-reactive protein	≥3 mg/L	n	22	31	20	39	35	50	54	251
		%	35.5	46.3	40.8	37.9	38.9	45.9	33.3	39.1

Abbreviations: BMI = body mass index; FAS = full analysis set; HbA_1c _= glycated hemoglobin; HDL = high density lipoprotein; Prev-AP = previous antipsychotic treatment cohort

* cutoffs as specified by laboratory

### MetS at Baseline

For both MetS definitions, NCEP-ATP III and AHA/NHLB, the differences between the cohorts with previous antipsychotic treatment were not statistically significant (Table 9). However, the Prev-None cohort had a significantly lower prevalence of MetS compared to any other Prev-AP cohort according to both definitions, except Pre-Risp (difference not significant).

**Table 9:** Prevalence of metabolic syndrome according to NCEP-ATP III and AHA/NHLB definitions by previous antipsychotic treatment at baseline, Prev-AP, FAS, N = 642

NCEP-ATP III				
**Cohort**	**N**	**n**	**%**	**95% CI**

Missing		4	0.6	-
Prev-Olz	62	30	48.4	35.5 to 61.4
Prev-Risp	66	25	37.9	26.2 to 50.7
Prev-Quet	49	23	46.9	32.5 to 61.7
Prev-Atyp	102	45	44.1	34.3 to 54.3
Prev-Typ	90	38	42.2	31.9 to 53.1
Prev-Comb	107	52	48.6	38.8 to 58.5
Prev-None	162	34	21.0	15.0 to 28.1
Total	638	247	38.7	34.9 to 42.6

**AHA/NHLB**				

Cohort	N	n	%	95% CI

Missing		4	0.6	-
Prev-Olz	62	30	48.4	35.5 to 61.4
Prev-Risp	66	28	42.4	30.3 to 55.2
Prev-Quet	49	25	51.0	36.3 to 65.6
Prev-Atyp	102	50	49.0	39.0 to 59.1
Prev-Typ	90	39	43.3	32.9 to 54.2
Prev-Comb	107	61	57.0	47.1 to 66.5
Prev-None	162	40	24.7	18.3 to 32.1
Total	638	273	42.8	38.9 to 46.7

Abbreviations: AHA/NHLB = American Heart Association/National Heart, Lung and Blood Institute

CI = confidence interval, FAS = full analysis set; NCEP-ATP III = National Cholesterol Education Program, Adult Treatment Panel 3^rd ^report; Prev-AP = previous antipsychotic treatment

### Development of MetS between baseline and endpoint at month-3

In the following, we report results for MetS according to AHA/NHLB-definition only, as both definitions are largely based on the same parameters; only the AHA/NHLB-definition additionally includes the treatment with antihypertensives, antidiabetics and lipid lowering drugs and was therefore regarded the more sensitive instrument.

At baseline, New-Typ had a significantly higher prevalence than New-Olz and New-Risp, but not compared to the other New-AP cohorts (differences lacked significance, see CIs in Table 10). At month-3 the MetS prevalence had increased from 44.3% to 49.6%; however, this change was not significant (95% CIs overlapping substantially). Comparing the New-AP cohorts, observed changes included minor changes, but none of these were statistically significant (Table 10).

**Table 10:** Prevalence rates of MetS according AHA/NHLB definition by new antipsychotic treatment, at baseline and after 3 months, (New-AP, CMD-set, N = 476)

Visit 1 (Baseline)				
**Cohort**	**N**	**n**	**%**	**95% CI**

New-Olz	206	79	38.4	31.7 to 45.4
New-Risp	69	24	34.8	23.7 to 47.2
New-Quet	33	18	54.6	36.4 to 71.9
New-Atyp	72	34	47.2	35.3 to 59.4
New-Typ	16	12	75.0	47.6 to 92.7
New-Comb	80	44	55.0	43.5 to 66.2
CMD-total	476	211	44.3	39.8 to 48.9

**Visit 2 (month-3)**				

Cohort	N	n	%	95% CI

New-Olz	206	93	45.2	38.2 to 52.2
New-Risp	69	34	49.3	37.0 to 61.6
New-Quet	33	16	48.5	30.8 to 66.5
New-Atyp	72	34	47.2	35.3 to 59.4
New-Typ	16	11	68.8	41.3 to 89.0
New-Comb	80	48	60.0	48.4 to 70.8
CMD-total	476	236	49.6	45.0 to 54.2

Abbreviations: AHA/NHLB = American Heart Association/National Heart, Lung and Blood Institute

CI = confidence interval, CMD = complete metabolic data; MetS = metabolic syndrome; NCEP-ATP III = National Cholesterol Education Program, Adult Treatment Panel 3rd report; New-AP = new antipsychotic treatment

Table 11 provides an overview on the change of the particular MetS-factors. Large standard deviations indicate a great variability of individual change in both directions. Looking at the median, however, little to no change was observed in waist-circumference, blood pressure, CRP, and HbA_1c. _There was an increase in median glucose values in all cohorts but New-Risp, and also in triglycerides with exception of the New-Typ and New-Comb. A decrease in the median HDL-cholesterol values was observed in all cohorts.

**Table 11:** Change of metabolic syndrome components by post-baseline cohort, CMD-set, New-AP cohorts

CMD-set		New-Olz	New-Risp	New-Quet	New-Atyp	New-Typ	New-Com	Total
	N	206	69	33	72	16	80	476
Waist (cm)	Mean	2.2	1.6	-1.4	-0.2	-1.2	0.8	1.1
	SD	7.9	5.8	3.5	5.3	4.3	6.0	6.7
	Median	1.0	0.0	0.0	0.0	0.0	0.0	0.0

Triglycerides (mkg/dL)	Mean	-4.1	35.2	23.5	-4.1	-7.3	-8.9	2.6
	SD	115.2	98.1	137.0	124.1	78.1	130.7	118.1
	Median	8.5	23.0	6.0	4.5	-17.0	-7.5	6.0

HDL (mg/dL)	Mean	-0.1	-1.8	0.6	-0.8	0.5	0.7	-0.3
	SD	9.2	11.1	10.4	8.7	6.0	9.5	9.5
	Median	-1.0	-1.0	-2.0	-2.0	-0.5	-0.5	-1.0

SBP (mmHg)	Mean	1.5	2.8	-2.8	-4.1	1.2	-2.0	-0.1
	SD	11.0	14.1	11.8	14.0	8.2	11.1	12.2
	Median	0.0	0.0	0.0	0.0	2.0	0.0	0.0

DBP (mmHg)	Mean	0.0	0.9	-0.4	-2.4	0.7	-1.3	-0.4
	SD	8.1	9.3	9.9	9.2	6.4	8.5	8.6
	Median	0.0	0.0	0.0	0.0	0.0	0.0	0.0

Glucose (mg/dL)	Mean	0.5	2.6	3.7	2.1	0.6	-4.4	0.4
	SD	26.4	30.4	65. 6	37.2	16.1	32.0	33.4
	Median	2.0	0.0	4.0	1.5	2.0	0.5	1.0

CRP (mg/L)	Mean	0.0	0.7	0.1	0.1	-2.7	-1.5	-0.2
	SD	4.6	7.3	1.6	4.8	10.5	8.9	6.1
	Median	0.0	0.0	0.0	-0.2	0.0	0.1	0.0

HbA_1c _(%)	Mean	0.0	-0.1	-0.1	0.0	-0.1	0.0	0.0
	SD	0.3	0.2	1.0	0.4	0.3	0.4	0.4
	Median	-0.1	-0.1	0.0	0.0	-0.1	0.0	0.0

Abbreviations: CRP = C-reactive protein; CMD = complete metabolic data; DBP = diastolic blood pressure; HbA_1c _= glycated hemoglobin, New-AP = new antipsychotic treatment cohort; SBP = systolic blood pressure; SD = standard deviation, Waist = waist circumference

### Factors associated with MetS (NCEP-ATP III -definition)

Factors found significantly associated with the presence of MetS in the multivariate logistic regression (CMD) were concomitant somatic disease (adjusted OR 4.09, p < 0.0001) and non-smoking (smoking vs. not, adjusted OR 0.53, p = 0.0098) at baseline. The same was observed at month-3, with an adjusted OR of 0.60 (p = 0.049) for smoking versus non-smoking, and a still negative, though not significant, effect of having any concomitant somatic disease (adjusted OR 1.83, p = 0.0796). Other factors associated with MetS at month-3 included male sex (female vs. male, OR 0.56, p = 0.0185), having a CRP ≥ 3 mg/L (adjusted OR of 2.00, p = 0.006), and receiving non-psychiatric concomitant medication (adjusted OR of 1.98, p = 0.059). In the baseline multivariate model the factors *CRP ≥3 mg/L *and *concomitant non-psychiatric medication *were eliminated during the multivariable forward selection process, though they showed significance in the univariate logistic regressions (CRP≥ 3 mg/L unadjusted OR of 1.68 [1.11;2.56], p = 0.015, concomitant non-psychiatric medication OR of 3.38 [2.14;5.31], p < 0.0001).

The sex effect did not demonstrate significance in univariate logistic regression (unadjusted OR female versus male of 0.82, p = 0.28).

An overview of factors associated with the presence of MetS is given in Table 12.

**Table 12:** Factors associated with MetS according to NCEP-ATP III criteria, results from univariate and multivariate logistic regression, (CMD- set, N = 476)

Univariate logistic regression Effect, Visit 1	Odds Ratio	95% CI	p-Value
Age	1.03	1.02 to 1.05	<.0001
Time since first symptoms (years)	1.02	1.00 to 1.04	0.0399
Concomitant somatic disease: Y vs. N	4.83	3.09 to 7.53	<.0001
Non-psychiatric co-medication: Y vs. N	3.38	2.15 to 5.31	<.0001
Smoking status: Y vs. N	0.61	0.42 to 0.89	0.0107
CRP ≥3 mg/L vs. normal value	1.68	1.11 to 2.56	0.0151
Prev-Comb vs. Prev-None	3.56	1.89 to 6.70	<.0001
Prev-Olz vs. Prev-None	2.91	1.40 to 6.05	0.0043
Prev-Atyp vs. Prev-None	3.27	1.72 to 6.24	0.0003
Prev-Quet vs. Prev-None	3.74	1.73 to 8.09	0.0008
Prev-Risp vs. Prev-None	2.62	1.27 to 5.39	0.0091
Prev-Typ vs. Prev-None	3.07	1.59 to 5.91	0.0008

**Effect, Visit 2**	Odds Ratio	95% CI	p-Value

Age	1.02	1.01 to 1.04	0.0042
Time since first symptoms (years)	1.03	1.01 to 1.04	0.0059
Concomitant somatic disease: Y vs. N No	3.98	2.57 to 6.19	<.0001
Non-psychiatric co-medication: Y vs. N No	2.67	1.71 to 4.16	<.0001
CRP ≥3 mg/L vs. normal value	2.36	1.58 to 3.51	<.0001
Prev-Comb vs. Prev-None	2.63	1.44 to 4.81	0.0017
Prev-Olz vs. Prev-None	2.63	1.30 to 5.33	0.0071
Prev-Atyp vs. Prev-None	2.07	1.11 to 3.85	0.0216
Prev-Quet vs. Prev-None	2.38	1.13 to 5.04	0.0232
Prev-Risp vs. Prev-None	2.16	1.08 to 4.33	0.0292
Prev-Typ vs. Prev-None	2.29	1.22 to 4.29	0.0098

**Multivariate logistic regression** **Effect, Visit 1**	Odds Ratio	95% CI	p-Value

Concomitant somatic disease: Y vs. N	4.09	2.37 to 7.06	<.0001
Smoking status: Y vs. N	0.53	0.32 to 0.86	0.0098

**Effect, Visit 2**	Odds Ratio	95% CI	p-Value

CRP ≥3 mg/L vs. normal value	2.00	1.22 to 3.30	0.0062
Non-psychiatric co-medication: Y vs. N No:	1.98	0.98 to 4.04	0.0588
Concomitant somatic disease: Y vs. N No	1.83	0.93 to 3.61	0.0796
Sex: female vs. male	0.56	0.34 to 0.91	0.0185
Smoking status at visit 2: Y vs. N	0.60	0.37 to 1.00	0.0488

Abbreviations: CI = confidence interval; CMD = complete metabolic data; CRP = ; C-reactive protein; MetS = metabolic syndrome; N = No; NCEP-ATP III = National Cholesterol Education Program, Adult Treatment Panel 3rd report; New-AP = new antipsychotic treatment cohort; Y = Yes

## Discussion

Baseline data showed that the study population comprised patients with a wide range of age and duration of disease. As patients could be either untreated or in need of a treatment switch, this study possibly included patients who received antipsychotic medications for years, but eventually had to be switched due to treatment-emergent adverse events or insufficient efficacy.

The percentages of patients with known concomitant hypertension (16.7%), lipid metabolism disorder (6.7%) and diabetes (5.6%) appeared moderate compared to numbers from German primary care patients (hypertension 31.6%, lipid metabolism disorder 23.4%, diabetes 9.4%) [29]. However, the vital signs and laboratory data collected at baseline revealed high blood pressure in 54.8%, increased triglycerides in 52.5% and increased blood glucose in 14.1% of the patients. This remarkable discrepancy emphasizes how important the actual monitoring of vital signs and blood values is in patients with schizophrenia, as seemingly, a large proportion of these patients were neither aware of their somatic health status nor adequately diagnosed and treated for cardiovascular risk factors.

Regarding baseline differences between the treatment groups (Prev-AP and New-AP), only two cohorts contrasted perceptibly from the others: One was the small (N = 16) group of New-Typ. These patients had clinically noticeable high mean values for BMI (32.3 kg/cm²), waist circumference (111.3 cm) and blood pressure (SBP/DBP 134.6/84.6 mmHG), and 12 of them (75%) actually met the criteria of MetS (AHA/NHLB). Though this cohort was too small for reliable statistical evidence, a possible explanation might be that these patients were switched/newly initiated on typical antipsychotics, because their metabolic and cardiovascular risk was already evident and these substances were assumed to have a lower risk of treatment-emergent metabolic adverse events. Though, in our study, the perception of lower risk of metabolic adverse events through typical antipsychotics was not supported by the baseline values found in the Prev-Typ cohort.

The other treatment cohort with noteworthy baseline values was Prev-None. These previously untreated patients showed numerically lower mean values for BMI, blood pressure, prevalence of somatic concomitant disease and practically all laboratory parameters than any other Prev-AP cohort, but had a comparatively higher symptom severity at baseline (mean CGI-S 4.2).

Apart from Prev-None, the Prev-AP cohorts did not contrast clearly with respect to baseline values; the highest percentages of patients with laboratory values out of normal range dispersed in different treatment groups for different parameters (see Table 8). This possibly reflects that changes in metabolic parameters may occur in patients treated with any antipsychotic medication, though these may differ in grade and type according to the properties of the respective substance and the patients' individual risk factors.

The prevalence of MetS in the FAS of 42.8% (AHA/NHLB definition) at baseline was comparable to the findings from the CATIE study, which reported a baseline MetS prevalence of 42.7% in an US-American sample of patients with schizophrenia [28].

The Prev-AP cohorts who had received some previous antipsychotic treatment showed no statistically significant differences in MetS-rates (AHA/NHBL). However, patients who entered our study untreated (Prev-None) had a baseline MetS prevalence of 24.7%, which was significantly lower than in any other cohort but Prev-Risp (42.4%, but overlapping CI). For comparison, Moebus et al. [30] reported a MetS prevalence rate of 28.6 ± 0.45% (AHA/NHLB criteria) in a cross-sectional sample of 33,502 primary care patients in Germany. Considering that Moebus' patients had a higher mean age than our study sample (53.0 ± 15.8 years in men and 50.9 ± 16.2 years in women versus 43.1 ± 13.1 and 47.3 ± 13.1 years, respectively, in our study), the prevalence of MetS in the Prev-None cohort appears to resemble the rates seen in primary care patients.

Considering the changes in MetS prevalence, the differences between baseline and month-3 lacked significance for all New-AP groups. Though, looking at the mean change of the particular MetS-components, a trend to increase was apparent in lipids, which could be a possible early predictor.

The results from logistic regression models at visit 2 indicate that the factors "*increased CRP*", "*concomitant somatic diseases*", and "*concomitant non-psychiatric medication*" increased the odds to develop MetS, while "*female sex*" and "*smoking*" decreased them. The factors "*concomitant somatic disease*" and "*concomitant non-psychiatric medication*" are in part comprised in the MetS definitons, and CRP is an established indicator of cardiovascular risk [31,32]. We did not expect, however, to find that smoking decreased the odds for MetS; this might possibly be an effect of the appetite reducing properties of nicotine [33].

Regarding the lower MetS-odds for women, data from the German general population [34] show women to have a lower incidence of cardiovascular and cerebrovascular events than men up to the age of 64, after which the respective rates converge (cardiovascular) or even become inverted (cerebrovascular). The review of cardiovascular risk factors in women by Evangelista and MacLaughlin [35], comprising international data published between 1990 and 2008, provided similar results. Considering the age structure of our study sample (FAS: mean age 45.2 years, Q1 36 years, Q3 54 years) our results fit well into the general picture.

They do, however, contradict the results from the CATIE study: McEvoy et al. [28] reports MetS-prevalences of 36.0% in men and 51.6% in women (fasting cohort, N = 689); the higher risk for MetS in women was a universal finding in all age groups, races and ethnicities. However, CATIE was a controlled clinical trial, so apart from country specific confounders as behavioral and dietary habits; possible selection bias might have impacted the results.

Several limitations of this study should be considered: As the study did not reach the required sample size, the analyses were underpowered, and therefore logistic regression models might have failed to detect all effects associated with MetS. Furthermore, the observational period of three months might have been too short to observe certain changes in metabolic status as e.g. development of insulin resistance or the processes leading eventually to increased CRP. Due to the observational design, treatment cohorts were defined post-hoc, depending on the actual case numbers treated with each antipsychotic, and compounds which were less frequently prescribed had to be grouped.

## Conclusions

Nevertheless, the MetS-rates found in this German sample of schizophrenia patients confirm the notion that MetS-prevalence is higher in patients with schizophrenia compared to the general population, with rates increasing with the duration of illness [36]. Even though three months seemingly were too short to retrieve statistically sound evidence on all possible risk factors, we observed an early increase of triglyceride levels. Our results once more emphasize how important the controlling of the patients' metabolic situation is in schizophrenia therapy [37,38] irrespective of antipsychotic medication.

## Competing interests

Susanne Kraemer, Anette Minarzyk, and Hans-Peter Hundemer are full-time employees of Lilly Deutschland GmbH. Thomas Forst and Daniel Kopf are members of an Eli Lilly advisory board and have received research funding from Eli Lilly.

## Authors' contributions

SK supported the conduct of the study and contributed to the data analysis, interpretation of data and writing of this report.

AM contributed to the data analysis, interpretation of data, and writing of this report.

HPH, DK and TF contributed to the study design, interpretation of data, and added scientific input to this report in form of comments. All authors contributed to and have approved the final manuscript.

## Pre-publication history

The pre-publication history for this paper can be accessed here:

http://www.biomedcentral.com/1471-244X/11/173/prepub
